# Lowering low-density lipoprotein cholesterol: from mechanisms to therapies

**DOI:** 10.1093/lifemeta/loac004

**Published:** 2022-05-20

**Authors:** Jie Luo, Jin-Kai Wang, Bao-Liang Song

**Affiliations:** College of Life Sciences, Hubei Key Laboratory of Cell Homeostasis, TaiKang Center for Life and Medical Sciences, TaiKang Medical School, Wuhan University, Wuhan, China; College of Life Sciences, Hubei Key Laboratory of Cell Homeostasis, TaiKang Center for Life and Medical Sciences, TaiKang Medical School, Wuhan University, Wuhan, China; College of Life Sciences, Hubei Key Laboratory of Cell Homeostasis, TaiKang Center for Life and Medical Sciences, TaiKang Medical School, Wuhan University, Wuhan, China

**Keywords:** cholesterol, low-density lipoprotein, statin, ezetimibe, proprotein convertase subtilisin/kexin type 9, atherosclerotic cardiovascular disease

## Abstract

Low-density lipoprotein (LDL) is the main carrier of cholesterol and cholesteryl ester in circulation. High plasma levels of LDL cholesterol (LDL-C) are a major risk factor of atherosclerotic cardiovascular disease (ASCVD). LDL-C lowering is recommended by many guidelines for the prevention and treatment of ASCVD. Statins, ezetimibe, and proprotein convertase subtilisin/kexin type 9 inhibitors are the mainstay of LDL-C-lowering therapy. Novel therapies are also emerging for patients who are intolerant to statins or respond poorly to standard treatments. Here, we review the most recent advances on LDL-C-lowering drugs, focusing on the mechanisms by which they act to reduce LDL-C levels. The article starts with the cornerstone therapies applicable to most patients at risk for ASCVD. Special treatments for those with little or no LDL receptor function then follow. The inhibitors of ATP-citrate lyase and cholesteryl ester transfer protein, which are recently approved and still under investigation for LDL-C lowering, respectively, are also included. Strategies targeting the stability of 3-hydroxy-3-methylglutaryl-coenzyme A reductase and cholesterol catabolism can be novel regimens to reduce LDL-C levels and cardiovascular risk.

## Introduction

Cholesterol is the most abundant steroid in human body. It can exist in the unesterified form and serve as a basic component of cell membranes, a precursor to other biologically active molecules, as well as a lipid moiety covalently linked to the Hedgehog and Smoothened proteins [1]. Cholesterol is also found esterified with long-chain fatty acids, forming cholesteryl esters (CEs) that store in the lipid droplets in the cell and the hydrophobic core of lipoproteins in the blood. Lipoprotein-carried CEs, together with free cholesterol embedded in the phospholipid monolayer of lipoproteins, represent the transportable form of cholesterol circulating throughout the body. Among all kinds of lipoprotein particles, low-density lipoproteins (LDLs) are primarily responsible for delivering cholesterol from the liver to other peripheral tissues. LDLs originate from liver-produced very low-density lipoproteins (VLDLs) as the latter travel through the bloodstream and their carried triglycerides are degraded by capillary endothelial cell-associated lipoprotein lipase (LPL). Cholesterol and CEs in LDLs therefore include those synthesized *de novo* in the liver as well as cholesterol obtained from the diets, which is packaged into VLDLs following hepatic uptake of chylomicrons. At the target organs such as the heart and muscle as well as the liver itself, LDLs are removed from the blood via receptor-mediated endocytosis, releasing cholesterol for structural and functional purposes.

The plasma level of LDL cholesterol (LDL-C) is an important index for cardiovascular health. According to the latest ESC/EAS guidelines, the values below 116 mg/dl are considered optimal for adults with low risk of cardiovascular events [2]. However, patients with familial hypercholesterolemia (FH)—an inherited lipid disorder caused mostly by mutations in the LDL receptor (*LDLR*) gene—can have plasma LDL-C levels several folds higher than normal individuals [3]. A direct consequence of LDL-C elevation is increased risks for atherosclerotic cardiovascular disease (ASCVD) [4], which is an umbrella term covering heart and blood vessel disorders resulted from LDL deposition and subsequent atherosclerotic plaque formation within the arterial intima. These plaques may develop silently over the years and rupture suddenly, resulting in catastrophic consequences such as ischemic stroke and myocardial infraction. Indeed, ASCVD is estimated to affect over 500 million people and accounts for 18.6 million deaths globally in 2019 [5]. It also poses enormous financial burdens on affected individuals and the whole society [6].

LDL-C lowering is recommended by the major clinical practice guidelines in primary and secondary prevention of ASCVD [2, 7]. Three widely prescribed LDL-C-lowering agents are statins, ezetimibe, and proprotein convertase subtilisin/kexin type 9 (PCSK9) antibodies, with statins being the first-line therapy and the other two added in the case of statin limitation or intolerance. On average, every 38.7 mg/dl decrease in LDL-C following statin and nonstatin therapies causes a more than 20% reduction in 5-year rate of major cardiovascular events [8]. Despite the huge success of the statins, there are still patients who fail to achieve LDL-C goals even with the combined treatments. Moreover, mutations in the *LDLR*, apolipoprotein B (*APOB*), and *PCSK9* genes, which are known to cause FH through affecting LDL clearance, account for merely 2.5% of cases of LDL-C elevation [9]. In the face of these challenges, however, both basic research and clinical translation advance rapidly, with several novel drugs and therapeutic strategies recently approved or currently tested.

**Figure 1:**
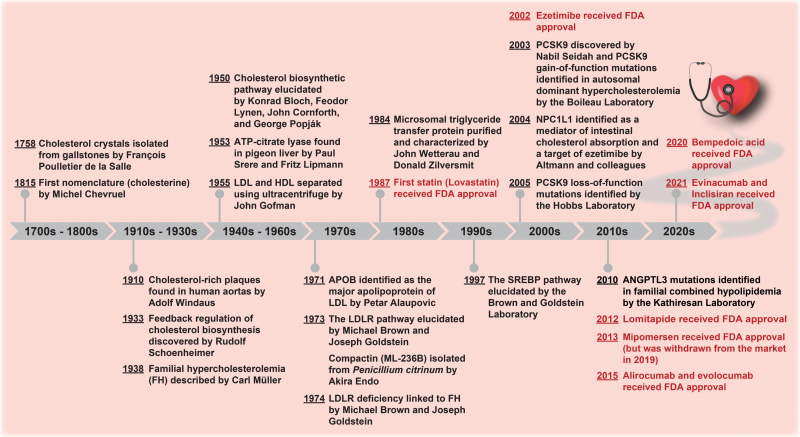
Timeline showing the milestones in the history of cholesterol and the development of LDL-C-lowering drugs. Drug launches are in red.

This review is intended to discuss the present and emerging LDL-C-lowering therapies (Fig. 1), focusing particularly on their mechanisms of action (Table 1). For the clinical efficacy of major LDL-C-lowering drugs, the readers are referred to several excellent reviews [10, 11] for details. Special strategies for homozygous FH patients who are usually unresponsive to standard regimens are included as well. The additional and conceptually promising LDL-C-lowering approaches are discussed in the end. Niacin, fibrates, and bile acid sequestrates are not mentioned here since their clinical use is declining.

**Table 1.** Treatments for lowering LDL-C.

Drug	Recommended dosing	Target	Mode of action	Mechanism of action
Statins	Intensity- and type-dependent, e.g.	HMGCR	Small molecule inhibitor	Inhibiting cholesterol biosynthesis and upregulating LDLR expression in the liver
	• Lova 20 mg (low intensity^a^);			
	• Atorva 10–20 mg (moderate intensity^b^), 40–80 mg (high intensity^c^);			
	• Rosuva 5–10 mg (moderate intensity), 20–40 mg (high intensity)			
Bempedoic acid	180 mg orally once a day	ACL	Small molecule inhibitor	Inhibiting cholesterol biosynthesis and upregulating LDLR expression in the liver
Ezetimibe	10 mg orally once a day	NPC1L1	Small molecule inhibitor	Inhibiting intestinal cholesterol absorption and upregulating hepatic LDLR expression
Alirocumab	75–150 mg subcutaneously every 2 weeks, or 300 mg subcutaneously every 4 weeks	PCSK9	Fully human monoclonal antibody	Blocking PCSK9-mediated LDLR degradation
Evolocumab	140 mg subcutaneously every 2 weeks, or 420 mg subcutaneously every 4 weeks	PCSK9	Fully human monoclonal antibody	Blocking PCSK9-mediated LDLR degradation
Inclisiran	284 mg subcutaneously initially, again at 3 months, and then every 6 months	PCSK9	siRNA	Blocking PCSK9-mediated LDLR degradation
Mipomersen^d^	200 mg subcutaneously once a week	APOB	ASO	Blocking APOB100 synthesis and VLDL production
Lomitapide	5 mg orally once a day for at least 2 weeks; and then increase to 10, 20, and 40 mg daily for at least 4 weeks; and up to the maximum dose of 60 mg daily	MTP	Small molecule inhibitor	Inhibiting the assembly and secretion of VLDL and chylomicron
Evinacumab	15 mg/kg body weight intravenously every 4 weeks	ANGPTL3	Fully human monoclonal antibody	Promoting LPL-mediated hydrolysis of triglycerides of VLDL and chylomicron, and derepressing EL-mediated hydrolysis of lipids of VLDL

Daily dose lowers LDL-C on average, by <30%.

Daily dose lowers LDL-C on average, by approximately 30% to <50%.

Daily dose lowers LDL-C on average, by approximately ≥50%.

Withdrawn from the market in 2019.

## Classical low-density lipoprotein cholesterol-lowering trios

The blood LDL-C levels are modulated by many factors such as hepatic cholesterol biosynthesis, cholesterol absorption from dietary and biliary sources, LDL clearance by LDLR-dependent or -independent mechanisms, interconversion between LDL and other lipoproteins, and cholesterol catabolism and excretion [12]. Blocking cholesterol biosynthesis and absorption and enhancing LDL removal from the blood underlie the effects of statins, ezetimibe, and PCSK9 inhibitors.

### Cholesterol biosynthesis and statins

**Figure 2:**
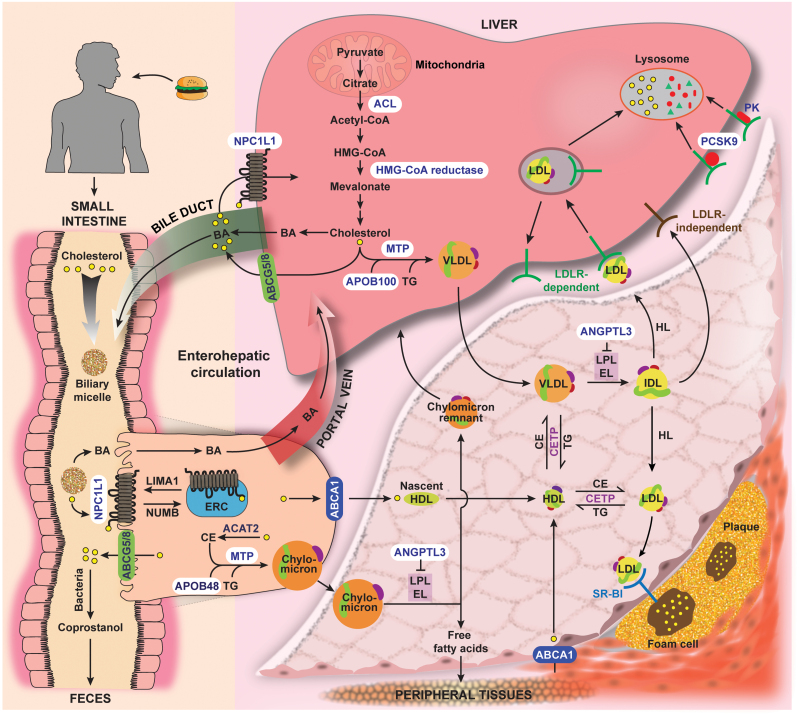
Major pathways of cholesterol metabolism in the body, with the pharmacological targets highlighted in white. Cholesterol in the body can be acquired from food or synthesized from acetyl-CoA in the liver. In the small intestine, dietary cholesterol and biliary cholesterol are emulsified by bile acid (BA) in the micelles, followed by NPC1L1-mediated internalization by enterocytes lining the brush border membrane of the intestine. This endocytic process involves the clathrin adaptor protein NUMB. Internalized cholesterol is first sent to the ERC and then to the ER (not shown), where it is converted to CE by ACAT2. Free cholesterol, CE, triglyceride (TG), and intestine-produced APOB48 are packaged, with the help of MTP, into chylomicrons that are secreted to the blood. NPC1L1 is recycled back to the surface for reuse by LIM domain and actin binding 1 (LIMA1). Excess free cholesterol can be exported to the intestinal lumen by the ABCG5/8 heterodimer, or to the blood by ABCA1, which leads to the formation of nascent HDL. About 50% of cholesterol in the intestine is excreted in the feces, either in the unmetabolized form or after conversion to coprostanol by intestinal bacteria. In the liver, acetyl-CoA for cholesterol biosynthesis is derived from citrate i.e. generated in the mitochondria, transported across the mitochondrial membrane and cleaved by ACL in the cytosol. The conversion of HMG-CoA to mevalonate by HMGCR is the rate-limiting step of cholesterol biosynthesis. Cholesterol, CE, and TG aggregate with liver-produced APOB100 to form VLDL. Excess cholesterol is excreted by ABCG5/8 into bile canaliculus. Biliary cholesterol can be reabsorbed by NPC1L1 on the canalicular membrane. Cholesterol can also give rise to BA that cycles between the liver and intestine multiple times a day. In the blood, lipoproteins undergo constant remodeling. Lipolysis of chylomicron and VLDL by LPL and probably EL give rise to chylomicron remnant and IDL, respectively. The activity of LPL and EL is blocked by ANGPTL3. The resultant free fatty acids are taken up by the peripheral tissues such as the heart, muscle, and adipose tissue, whereas chylomicron remnant and IDL are cleared from circulation. IDL can be taken by the liver directly without involving LDLR, or convert to LDL through hepatic lipase (HL)-mediated lipolysis. LDL binds LDLR on the apical surface of liver and is internalized, finally releasing the carried cholesterol in the lysosome. LDLR can be targeted to lysosomal degradation by liver-secreted PCSK9 and plasma prekallikrein (PK). The accumulation and oxidization of LDL in the intima of arteries drive the formation of foam cells and subsequent atherosclerotic plaques. SR-BI, scavenger receptor class B type I. As for HDL metabolism, nascent HDL incorporates free cholesterol exported by ABCA1 from the peripheral tissues and finally becomes mature HDL. Mature HDL can exchange CE with VLDL or LDL for TG in the opposite direction by the action of CETP. HDL also receives excess cholesterol from peripheral tissues and contributes to reverse cholesterol transport (not shown).

The liver is the primary site of cholesterol biosynthesis. The formation of cholesterol begins with acetyl-coenzyme A (CoA) (Fig. 2). Two molecules of acetyl-CoA first condense to form acetoacetyl-CoA, which is then condensed with another acetyl-CoA to produce 3-hydroxy-3-methylglutaryl-CoA (HMG-CoA). In the next step, HMG-CoA undergoes NADPH-dependent reductions to yield mevalonate. This reaction, catalyzed by HMG-CoA reductase (HMGCR), serves as the primary rate-limiting step and the most important site of regulation in cholesterol biosynthesis. The generated mevalonate is further metabolized in a series of reactions, producing farnesyl pyrophosphate that situates at the branch point of cholesterol biosynthetic pathway. The condensation of two molecules of farnesyl pyrophosphate makes squalene and commits the cell to cholesterol production rather than giving rise to nonsterol isoprenoids such as geranylgeraniol. Squalene through oxidation and cyclization is next converted to lanosterol, the first steroid intermediate in the pathway. From lanosterol, the biosynthetic pathway is split into two, with the Bloch pathway beginning with lanosterol and the Kandutsch–Russell pathway with 24,25-dihydrolansterol generated from lanosterol via side chain reduction. Through a series of steroid ring modifications that run in parallel along both pathways, cholesterol is generated from the Kandutsch–Russell pathway directly as well as from side chain reduction of the last intermediate in the Bloch pathway. Many tissues including the liver utilize a hybrid of both pathways to synthesize cholesterol [13].

**Figure 3:**
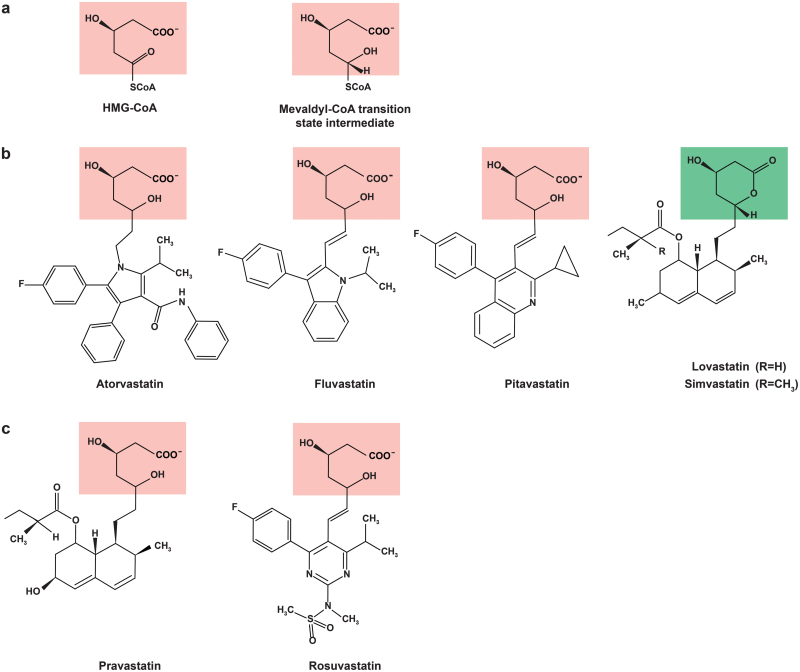
Chemical structures of HMG-CoA, mevaldyl-CoA transition state intermediate, and seven commercially available statins. Statins contain either the dihydroxyheptanoic acid unit (red) that resembles the endogenous substrate HMG-CoA or mevaldyl-CoA transition state intermediate as shown in (a), or the lactone ring (green) that opens upon hydrolysis. (b) Lipophilic statins. (c) Hydrophilic statins.

The LDL-C-lowering effects of statins are primarily attributed to their inhibition of cholesterol biosynthesis via HMGCR. At present, seven different statins are available on the market, all comprising a ring system with an acid or lactone moiety and polar or nonpolar substituents (Fig. 3). The dihydroxyheptanoic acid unit, which is readily available in five statins (atorvastatin, fluvastatin, pitavastatin, pravastatin, and rosuvastatin), has a structural resemblance to the endogenous substrate HMG-CoA and the mevaldyl-CoA transition state intermediate (Fig. 3), thereby mediating the binding of statins to the active site of HMGCR [14]. Lovastatin and simvastatin are administered as an inactive lactone (Fig. 3) and require hydrolysis by carboxylesterases in the body for activation [15]. The other additions to the ring system contribute to solubility and pharmacokinetics of different statins [16]. Those with polar substituents (e.g. rosuvastatin) are hydrophilic and enter the cells via carrier-mediated mechanisms, which minimize their effects at extrahepatic sites. By contrast, lipophilic statins (e.g. atorvastatin and lovastatin) owing to the presence of nonpolar substituents can diffuse nonselectively across the cell membranes and tissues and end up widely distributed. Also as a result of solubility, hydrophilic statins are eliminated largely unchanged whereas lipophilic ones are broken down by membrane-bound cytochrome P450 superfamily of enzymes.

In addition to inhibiting HMGCR activity, statins can reduce LDL-C levels by upregulating the expression of LDLR proteins. This effect is mediated by the sterol regulatory element-binding protein (SREBP) 2 pathway, which is activated by cholesterol depletion following statin treatment. SREBP2 is a member of the SREBP family proteins and functions as the master transcriptional regulator of cholesterol biosynthesis and uptake. It is synthesized as an inactive precursor and interacts with SREBP-cleavage activating protein in the endoplasmic reticulum (ER) membrane. Under low cholesterol levels, the SREBP/SREBP-cleavage activating protein complex is translocated in coatomer II vesicles from the ER to the Golgi, where the SREBP precursor undergoes a two-step cleavage and liberates the N-terminal fragment that finally enters the nucleus and activates the transcription of target genes, including those encoding cholesterogenic enzymes, LDLR, PCSK9, and many others. The resultant increase in LDLR proteins on the cell surface accounts for enhanced LDL clearance from the blood, with the detailed mechanism discussed later.

The dose of a statin that can reduce LDL-C by ≥50% is a high-intensity regimen [17]. Rosuvastatin and atorvastatin are two most potent and commonly prescribed statins and can be used up to 20–40 and 40–80 mg/day, respectively (Table 1). However, high doses of statins may be associated with side effects including muscle toxicity. About 8%–9% of primary or secondary prevention patients are intolerant to statins [18]. To reach optimal LDL-C levels, nonstatin agents, as those discussed below, are often required as add-on therapy, especially in patients with primary (heterozygous familial and nonfamilial) hypercholesterolemia.

### Cholesterol absorption and ezetimibe

Dietary cholesterol is an important source of cholesterol carried in LDL. Cholesterol in food is mostly unesterified and often mixed with triglycerides. Once reaching the small intestine lumen, cholesterol, together with other fats, is solubilized by bile acids in the form of mixed micelles and transports across the water layer to the brush border of the small intestine [19]. At the apical surface of enterocytes, cholesterol is taken up by an active pathway involving Niemann-Pick type C (NPC)1-like 1 (NPC1L1) [20], which is a membrane protein with 3 extracellular domains (N-terminal domain, middle domain, and cysteine-rich domain), 13 transmembrane segments, and a short cytosolic tail (Fig. 2). NPC1L1 is homologous to NPC1 that mediates the egress of LDL-derived cholesterol from the lysosomes [21]. As suggested by the structural and functional assays, NPC1L1-dependent cholesterol uptake is a multistep process involving vesicular transport from the plasma membrane (PM) to the endocytic recycling compartment (ERC) [22]. To ensure efficient uptake, NPC1L1 binds cholesterol via the extracellular N-terminal domain and promotes the formation of cholesterol-enriched and flotillins-containing membrane microdomains [23]. Cholesterol binding to NPC1L1 causes the dissociation of its C-terminal tail from the PM, exposing the YVNxxF internalization signal for the recruitment of NUMB and clathrin [24]. The invaginated membranes then pinch off from the PM, forming clathrin-coated vesicles that migrate along actin filaments toward the ERC, which is a perinuclear region containing cholesterol-enriched tubular and vesicular membranes [25, 26]. From the ERC, cholesterol is further passed to the ER, where it is converted to CEs by acyl-CoA:cholesterol acyltransferase 2 (ACAT2) and packaged into chylomicrons for secretion into the lymphatic system and then the plasma. Intracellular cholesterol can also be pumped across the apical membrane by ATP-binding cassette (ABC) transporter superfamily G members 5 and 8 (ABCG5/8) to the intestinal lumen, or across the basolateral membrane by ABC transporter superfamily A member 1 (ABCA1) to plasma apolipoprotein A–I for the generation of nascent high-density lipoprotein (HDL) particles (Fig. 2). Ablation of both ACAT2 and ABCA1 can reduce intestinal cholesterol absorption [27]. In response to cholesterol decrease in the ERC, NPC1L1 is recycled back to the PM by interacting with LIMA1, myosin Vb, and the small GTPase CDC42 [28–30].

Bile by providing at least twice as much cholesterol as normal diets represents another significant input for intestinal cholesterol [19]. Biliary cholesterol is excreted from the liver, where hepatocytes synthesize cholesterol as described in the earlier section and release the molecule from the canalicular membrane via the ABCG5/8 heterodimer (Fig. 2), following the excretion of bile acids that are in fact generated from cholesterol in hepatocytes as well [31]. This portion of cholesterol is assembled into micelles, mobilizes from the liver to the gallbladder and, upon food ingestion, is expelled to the intestinal lumen, finally mixing with dietary cholesterol for NPC1L1-dependent endocytosis. In humans, cholesterol in bile can be reabsorbed by the liver via NPC1L1 on the canalicular membrane (Fig. 2).

Ezetimibe inhibits cholesterol (re)absorption by targeting both intestinal and hepatic NPC1L1 protein [32, 33]. Several recent high-resolution cryo-electron microscopy studies reveal the presence of ezetimibe or its analog within the lumenal domains of NPC1L1 [34–36]. Ezetimibe has been suggested to prevent cholesterol binding to NPC1L1 [34], block cholesterol movement from the N-terminal domain to the transmembrane domain [35, 36], as well as disrupt the NPC1L1–flotillin association and thus the formation of cholesterol-enriched membrane microdomains [23]. This results in reduced cholesterol absorption from the small intestine and decreased formation of chylomicrons that normally supply the liver with dietary and biliary cholesterol. Together with attenuated hepatic cholesterol reabsorption that indirectly increases cholesterol excretion, ezetimibe decreases the cholesterol pool in the liver, thereby reducing VLDL production while inducing a compensatory upregulation of hepatic LDLR protein that leads to enhanced LDL-C clearance from the blood. Of note, ezetimibe and its glucuronide metabolite can repeatedly circulate between the small intestine and liver, with a half-life of about a day [37], which sustains the inhibitory effects on NPC1L1.

Ezetimibe is recommended to use at a dose of 10 mg daily by many guidelines (Table 1). Adding ezetimibe to statins can cause additional LDL-C reductions, without raising safety concerns, compared with statin monotherapy at both low and high doses [38].

### Low-density lipoprotein uptake and proprotein convertase subtilisin/kexin type 9 inhibitors

Compared with cholesterol biosynthesis and absorption that contribute to LDL generation, LDL uptake by tissue cells negatively regulates blood LDL-C levels. This process is mainly mediated by LDLR and occurs primarily in the liver [39] (Fig. 2). Akin to NPC1L1-mediated cholesterol absorption, LDLR binds APOB on the surface of LDL particle via the extracellular domain and recruits ARH (autosomal recessive hypercholesterolemia) and Disabled-2 as endocytic adaptors via the NPxY endocytic motif in the cytoplasmic tail [40, 41], thereby initiating clathrin-dependent endocytosis of the LDL/LDLR complex. These two, however, separate as LDLR undergoes a conformational change in the acidic endosomes. LDL is further delivered to the late endosomes and lysosomes to release the carried cholesterol, whereas LDLR is recycled to the surface to mediate the uptake of another LDL particle. The whole process from LDLR binding to LDL to its return to the surface is estimated to be 10 min [42, 43], which allows one LDLR to internalize hundreds of LDL particles during its life span of ~20 h. In the lysosomes, NPC1 and NPC2 proteins function in concert to deliver cholesterol from the lumen to the lysosomal membrane [44, 45], from where cholesterol is transported to the PM or other organelles by vesicular and nonvesicular mechanisms [46, 47].

PCSK9 is a secreted serine protease mainly produced by the liver and functions as a key post-translational regulator of LDLR. It can directly bind LDLR at the epidermal growth factor precursor homology domain A [48], which immediately follows the LDL-binding domain, and is then internalized with LDLR in clathrin-coated pits (Fig. 2). However, different from LDL and LDLR that dissociate in the endosomes, low pH actually increases PCSK9 binding to LDLR [49, 50], which prevents LDLR from undergoing a conformational change i.e. a prerequisite for its recycling to the surface [51]. Therefore, LDLR together with PCSK9 is targeted to the lysosomes for degradation [52, 53]. In addition to reducing LDLR proteins on the cell surface, PCSK9 prior to secretion can direct newly synthesized LDLR to the lysosomes for degradation as well [54]. A number of naturally occurring PCSK9 mutations have been identified in individuals with elevated or reduced levels of LDL-C, such as S127R and D374Y that increase PCSK9 affinity for LDLR [55], as well as G236S and S462P that reduce PCSK9 secretion by sequestering it in the ER [56, 57].

Inhibition of PCSK9-induced LDLR degradation is a third clinical approach for LDL-C lowering. This is particularly important considering that PCSK9 as a transcriptional target of SREBP2 is co-upregulated with LDLR following cholesterol depletion [58]. Therefore, increased plasma PCSK9 levels can limit the efficacy of statins and ezetimibe on lowering LDL-C [59–61]. Various strategies for PCSK9 inhibition have been proposed, among which include the injectable monoclonal antibodies (mAbs) [62]. Evolocumab and alirocumab are two fully human PCSK9 mAbs approved by the US Food and Drug Administration (FDA) and the European Medicines Agency in 2015 for use in high-risk patients requiring additional LDL-C reduction (Fig. 1). Following subcutaneous injections, evolocumab and alirocumab circulate into the bloodstream and bind with high affinity to PCSK9, preventing PCSK9 from interacting with LDLR and subsequent lysosomal degradation of LDLR. By this way, both LDLR on the surface of hepatocytes and LDLR clearance from the blood are increased. Two landmark randomized controlled trials, the FOURIER trial and the ODYSSEY OUTCOMES trial, show that evolocumab and alirocumab reduce LDL-C levels by about 60% and the relative risk of recurrent cardiovascular event by 15% on top of statin therapy in patients with established ASCVD [63, 64]. However, the high costs of evolocumab, which requires administration at a dose of 140 mg every 2 weeks or 420 mg every month, and alirocumab, which requires administration at a dose of 75 mg every 2 weeks or maximally 150 mg or maximally if LDL-C >70 mg/dl (Table 1), may pose a limit to their widespread use and patient adherence.

Another way to inhibit the action of PCSK9 on LDLR is to reduce its intracellular production using the small interfering RNA (siRNA). Inclisiran is a chemically synthesized siRNA duplex with the sense strand conjugated to triantennary *N*-acetylgalactosamine carbohydrates (GalNAc), which by binding to asialoglycoprotein receptors on the surface of hepatocytes confer liver-specific delivery [65], and the antisense strand targeting human PCSK9 mRNA. Once entering hepatocytes, two strands are bound and unwound by the RNA-induced silencing complex. The antisense strand then guides the RNA-induced silencing complex to PCSK9 mRNA and blocks its translation to protein. Inclisiran has received its initial approval by the end of 2020 and 2021 in Europe and USA, respectively, as an add-on therapy to further lower LDL-C (Fig. 1). Compared with PCSK9 mAbs with frequent injections, inclisiran lasts longer and a single subcutaneous dose of 284 mg at Day 1, Day 90, and then every 6 months are recommended (Table 1). Although siRNA-mediated silencing in principle should be more thorough than mAb neutralization to ablate PCSK9, the endosomal entrapment and lysosomal degradation may prevent siRNAs from reaching their targets [66]. Also, the efficacy of inclisiran on major cardiovascular outcomes needs to be assessed. The long-term safety of PCSK9 inhibition should be monitored since PCSK9 deficiency is recently found to impair cardiac function [67].

## Low-density lipoprotein cholesterol lowering in homozygous familial hypercholesterolemia patients

Homozygous FH occurs at an estimated prevalence of 1 in 300,000 and is characterized by plasma LDL-C levels commonly greater than 500 mg/dl in adults and 420 mg/dl in children [68]. The presence of cutaneous xanthomas since infancy is a very likely indicative of homozygous FH. If untreated, it can lead to fatal cardiovascular outcomes early in life. Most homozygous FH patients carry loss-of-function mutations in both alleles of the *LDLR* gene. So far, more than 2000 FH-causing variants of the *LDLR* gene have been identified (https://databases.lovd.nl/shared/genes/LDLR). Loss-of-function mutations in *APOB* and *ARH* and gain-of-function mutations in *PCSK9* can also cause homozygous FH but are less frequently encountered.

Lowering LDL-C in homozygous FH patients is challenging because they manifest poor or even no responses to statins, ezetimibe, and PCSK9 mAbs, which, in part or entirely, act by increasing LDLR levels and thus LDLR-mediated LDL uptake. LDLR-independent treatments are therefore required for homozygous FH patients and can benefit those with primary hypercholesterolemia as well.

### Very low-density lipoprotein production and mipomersen and lomitapide

VLDL is the precursor to LDL. The assembly of VLDL occurs in the ER lumen of hepatocytes, starting with the cotranslational addition of small amounts of phospholipids, triglycerides, and CEs to APOB100, which is the fully translated form of APOB specifically produced by the human liver (Fig. 2). The CEs are formed via the esterification of free fatty acids with cholesterol by ACAT2. This partial lipidation of APOB100 is facilitated by the ER-resident enzyme microsomal triglyceride transfer protein (MTP) [69], and results in stabilization of the nascent APOB100 protein and formation of a primordial VLDL particle. TMEM41B-mediated flipping of phospholipids from the cytosolic leaflet to the lumenal leaflet of the ER membrane supports the surface expansion of VLDL [70, 71]. The premature VLDL then undergoes a post-translational lipidation in the ER, en route to and in the Golgi, and acquires triglycerides from the ER or cytosolic lipid droplets as well as CEs. The cell death activator CIDEB and the transmembrane 6 superfamily member 2 have been shown to function in this bulk lipidation process [72–74]. The transmembrane 6 superfamily member 2 also plays a role in stabilizing APOB100 [75]. Beyond lipidation, VLDL maturation also includes glycosylation of APOB100 and recruitment of other apolipoproteins in the Golgi lumen [76]. The mature VLDL finally leaves the Golgi in transport vesicles and is secreted into the circulation.

Chylomicron is assembled and secreted by the small intestine in a more or less similar way to VLDL [77]. However, the essential structural protein of chylomicron is APOB48, a truncated form corresponding to N-terminal 48% of APOB100 as a result of intestine-specific, apobec-1-mediated mRNA editing [78]. APOB48 is first lipidated by MTP to form a primordial chylomicron particle, which, after being loaded with additional triglycerides, phospholipids, and CEs, becomes mature and is eventually secreted (Fig. 2).

Mipomersen is a human APOB mRNA-targeting antisense oligonucleotide (ASO) containing phosphorothioate linkages and 2ʹ-*O*-methoxyelthyl-modified nucleotides at both ends, which confer increased RNA affinity and resistance to nucleases [79]. It can bind to the coding region of cognate APOB mRNA via complementary base pairing and direct the latter for ribonuclease H-mediated degradation. Mipomersen is primarily distributed to the liver instead of the intestine after subcutaneous administration [80], thereby selectively inhibiting the synthesis of hepatic APOB100 protein and, consequently, that of APOB100-containing lipoproteins such as VLDL and LDL (Table 1).

Lomitapide is an orally administered small molecule that binds MTP and inhibits its function in transferring lipids onto nascent APOB100 and APOB48 proteins in hepatocytes and enterocytes, respectively. This results in reduced production (and thus secretion) of VLDLs and chylomicrons and ultimately decreases LDL levels in the plasma (Table 1).

Both mipomersen and lomitapide showed significant LDL-C-lowering efficacy in the phase 3 studies involving homozygous FH patients [81–84]. However, the other side of impairing lipoprotein assembly is the accumulation of triglycerides in the liver for mipomersen and in both the liver and the intestine for lomitapide. Increases in hepatic fat content and transaminase levels are the side effects of mipomersen and lomitapide. Mipomersen is also associated with injection-site reactions and influenza-like symptoms, whereas lomitapide is with gastrointestinal discomfort. Of note, since lomitapide is a substrate and an inhibitor of cytochrome P450 3A4 in the liver [85], drug–drug interactions should be considered when it is co-administered with lipophilic statins. In early 2013, mipomersen was approved by FDA for treating patients older than 12 years with homozygous FH (Fig. 1). However, it was withdrawn from the market in 2019 due to liver toxicity. Lomitapide as a therapy for homozygous FH has been approved by the regulatory agencies in more than 30 countries (Fig. 1).

### Lipoprotein metabolism and angiopoietin-like protein 3 inhibition

Lipoproteins in circulation are constantly remodeled and metabolized. Hydrolysis of the carried lipids is crucial for interconversion and catabolism of plasma lipoproteins. Three members of the triglyceride lipase family—LPL, hepatic lipase, and endothelial lipase (EL)—play important roles in lipoprotein lipolysis. LPL is produced by the extrahepatic cells and translocated from the interstitial space to the surface of capillary endothelium, where it hydrolyzes triglycerides in the hydrophobic core of VLDLs and chylomicrons (together called triglyceride-rich lipoproteins, TRLs) [86]. This liberates free fatty acids for utilization in muscle and heart tissues or storage in adipose tissue, as well as converts VLDLs and chylomicrons into smaller but denser particles, namely intermediate-density lipoproteins (IDLs) and chylomicron remnants, respectively (Fig. 2). Hepatic lipase, a liver-expressed lipolytic enzyme, is responsible for hydrolyzing triglycerides and phospholipids present in IDLs, chylomicron remnants and HDLs [87]. Hepatic lipase-mediated lipolysis of IDLs generates LDLs. EL is a predominant phospholipase synthesized by the endothelial cells of vascularized tissues and, together with LPL, promotes triglyceride hydrolysis and clearance of TRLs [88], and together with hepatic lipase, regulates the remodeling and catabolism of HDLs [89]. All three lipases are also implicated in mediating the uptake of lipoprotein particles independent of their lipolytic activity [87, 90, 91]. Other than lipolysis, triglycerides in TRLs can be transferred to LDLs and HDLs in exchange for CEs transferred from HDLs to TRLs and LDLs by cholesteryl ester transfer protein (CETP) [92].

Angiopoietin-like protein 3 (ANGPTL3) is a key determinant of plasma lipid levels. Once synthesized by the liver, it circulates throughout the body, binding and inhibiting the activity of LPL. This suppressive effect is greatly enhanced when ANGPTL3 forms a complex with ANGPTL8, another member of the angiopoietin growth factor family whose production is activated by refeeding [93, 94]. ANGPTL3 can also inhibit EL independently of ANGPTL8 [95, 96]. The inhibition of LPL-mediated triglyceride hydrolysis by ANGPTL3 explains the extremely low plasma triglyceride levels seen in individuals carrying the loss-of-function mutations in *ANGPTL3* [97]. These patients also have lower plasma levels of LDL-C and reduced risk for ASCVD than noncarriers; however, the mechanisms by which ANGPTL3 deficiency decreases LDL-C still remain unclear. Conflicting results on reduced secretion of hepatic VLDL triglyceride but not APOB100 or vice versa have both been reported [98–100]. Depletion of ANGPTL3 was also found to increase the expression of LDLR and LDLR-related protein 1 and the uptake of LDL and VLDL [99]. However, even when LDLR is absent, ANGPTL3 ablation can still reduce LDL-C levels [101, 102], through derepressing the role of EL in hydrolyzing phospholipids of VLDL, which results in the generation of remnant particles that are efficiently cleared from circulation [101]. This LDLR-independent lowering of LDL-C by ANGPTL3 deficiency represents an additional therapeutic option for the treatment of homozygous FH.

The current approaches to inhibit ANGPTL3 range from mAbs to ASO and siRNAs, all of which aim to block the activity or expression of ANGPTL3. Evinacumab is a fully human monoclonal antibody against ANGPTL3 and the only FDA-approved ANGPTL3 inhibitor for treating homozygous FH patients aged 12 years and older, as an adjunct to other lipid-lowering regimens (Fig. 1 and Table 1). In the preclinical trials, evinacumab reduces LDL-C levels by a mean of more than 40% in homozygous FH patients and more than 50% at the maximum dose in refractory hypercholesterolemia patients, most of which were already on statins, ezetimibe, PCSK9 inhibitors, and/or lomitapide [103–105]. Vupanorsen and ARO-ANG3 are GalNAc-conjugated ASO and siRNA, respectively, targeting ANGPTL3 mRNA in the liver. Unfortunately, the development of vupanorsen as a therapy for dyslipidemia and hypertriglyceridemia was terminated by Pfizer in January 2022, because the magnitude of reductions in non-HDL-C and triglycerides, albeit statistically significant, was insufficient to support further development. The association of vupanorsen with liver fat accumulation and toxicity also raises concerns. ARO-ANG3 demonstrated a dose-dependent lowering effect of LDL-C in a study involving 17 heterozygous FH patients [106]. The phase 2 study evaluating the safety and efficacy of ARO-ANG3 in homozygous FH patients (NCT05217667) will begin this June.

## Additional low-density lipoprotein cholesterol-lowering therapies

Two new kinds of agents, the recently approved bempedoic acid and currently evaluated anacetrapib and obicetrapib, also demonstrate a great efficacy on LDL-C lowering.

### Acetyl-CoA production and bempedoic acid

Acetyl-CoA is the substrate for cholesterol and fatty acid biosynthesis in the cell. Ample amounts of acetyl-CoA are generated in the mitochondria from pyruvate and fatty acid oxidation but cannot cross the mitochondrial membrane directly. To solve this problem, acetyl-CoA is first condensed with oxaloacetate in the mitochondria, forming citrate that can transport across the mitochondrial membrane, and is then regenerated in the cytosol by ATP-citrate lyase (ACL), an enzyme mainly expressed in the liver and white adipose tissue [107]. Acetyl-CoA next enters the cholesterol biosynthetic pathway involving HMGCR (Fig. 2).

Bempedoic acid lowers LDL-C levels through inhibiting cholesterol biosynthesis and enhancing the expression of LDLR. However, unlike statins that act on HMGCR, bempedoic acid targets ACL. Administered as a prodrug, bempedoic acid is converted into the active CoA thioester form by very long-chain acyl-CoA synthetase-1 i.e. most abundantly expressed by the liver [108]. The inhibition of ACL by bempedoyl-CoA reduces the production of acetyl-CoA and cholesterol in the downstream, thus leading to the activation of the SREBP2 pathway and increases in LDLR expression and LDL clearance (Table 1).

As revealed by 4 phase 3 CLEAR trials, bempedoic acid effectively reduces plasma LDL-C by 17%–18% in patients with high cardiovascular risk receiving a maximally tolerated statin and by 24% in patients with statin intolerance [109–113]. A fixed-dose combination of bempedoic acid (180 mg) and ezetimibe (10 mg) further lowers LDL-C compared with monotherapy [114]. Due to the absence of activating enzyme in the skeletal muscle, bempedoic acid has not been associated with muscle-related adverse events as seen with statin therapy. However, the concomitant use of bempedoic acid with simvastatin >20 mg or with pravastatin >40 mg increases statin exposure and risk of myopathy. As an orally taken, once-daily nonstatin alternative, bempedoic acid was approved in 2020 by FDA for use in patients with heterozygous FH or established ASCVD who are already on maximally tolerated statins but require additional LDL-C lowering (Fig. 1). In Europe, bempedoic acid is allowed to be used in people intolerant to statins or for whom a statin is contraindicated.

### Cholesteryl ester transfer protein inhibitors

CETP is a plasma glycoprotein produced mainly by the liver and binds preferentially to HDLs [115]. By exchanging CEs and triglycerides between HDLs and APOB-containing lipoproteins, CETP facilitates the formation of triglyceride-enriched HDLs and CE-enriched VLDL and LDL (Fig. 2). Triglycerides and phospholipids of HDLs are then hydrolyzed by hepatic lipase and EL, resulting in destabilization of HDL particles and dissociation of apolipoprotein A–I i.e. eventually cleared by the kidney; whilst CEs transferred from HDLs are delivered back to the liver for elimination in the bile and ultimately the feces [116]. CETP inhibition slows HDL catabolism, reduces CE transfer from HDLs to TRLs and LDLs, and increases LDL uptake by LDLR [117], leading to an increase in HDLs and a decrease in LDLs. Together with the early findings that humans with genetic deficiency of CETP had elevated levels of HDL and decreased levels of LDL [118, 119], the development of CETP inhibitors as a potential treatment of cardiovascular disease has garnered great interest.

A total of five CETP inhibitors have been developed so far, with raising plasma HDL levels being the primary focus initially. The first three were abandoned, due either to increases in cardiovascular events and deaths (torcetrapib) [120], or to the lack of effectiveness on reducing cardiovascular events (dalcetrapib and evacetrapib)[121, 122]. These failures for a long time posed uncertainty about CETP as a worthy therapeutic target, but were later attributed to compound-related issues including the off-target effects, weak LDL-C-lowering potency, and study design [117, 123]. In fact, a Mendelian randomization analysis involving over a million participants revealed that variants in the *CETP* gene that caused low CETP activity were associated with higher HDL-C levels, lower LDL-C and APOB levels, and a reduced risk of cardiovascular events [124]. Notably, CETP variant-associated cardiovascular risk was lessened in a similar magnitude to risks associated with the *HMGCR*, *NPC1L1*, and *PCSK9* variants per unit reduction in LDL-C. Anacetrapib is the second generation of CETP inhibitor capable of reducing LDL-C levels by enhancing LDL-APOB100 clearance [125]. Addition of anacetrapib to patients with ASCVD who received intensive atorvastatin therapy increased HDL level by 104% and decreased non-HDL level by 18% [126]. The incidence of major coronary events was reduced by 9% during a mean follow-up of 4.1 years and by further 20% in the extended mean follow-up of 2.2 years [127]. Obicetrapib is the most recent CETP inhibitor showing a much stronger potency on lowering LDL-C in the phase 2 study [128]. The phase 3 BROADWAY trial to evaluate the efficacy, safety, and tolerability of obicetrapib as an adjunct to diet and maximally tolerated lipid-lowering therapy for 365 days in patients with heterozygous FH and/or established ASCVD was just initiated (NCT05142722). If successful, obicetrapib will become a new LDLR-independent treatment for patients intolerant to statins.

## Future on low-density lipoprotein cholesterol lowering

With fast advances in technology and basic science, promising therapeutic options for lowering LDL-C are growing rapidly. This section focuses on two conceptually attractive approaches that can be harnessed to lower LDL-C, one to block cholesterol biosynthesis via HMGCR degraders, and the other to enhance cholesterol catabolism via intestinal microbiota.

### Cholesterol biosynthesis revisited and HMG-CoA reductase degraders

HMGCR is an ER-anchored transmembrane protein with a catalytic domain protruding into the cytosol [129], to which statins bind [14]. As the primary rate-limiting enzyme, HMGCR is modulated by different mechanisms operating at multiple levels [130]. The protein stability of HMGCR is negatively regulated by oxysterols derived from cholesterol, such as 25-hydroxycholesterol and 27-hydroxycholesterol, and sterol intermediates in the mevalonate pathway, such as lanosterol and 24,25-dihydrolansterol, as well as geranylgeraniol [131–134]. The *HMGCR* gene is a downstream target of the SREBP2 pathway. Hence, following HMGCR inhibition by statins, the production of sterols and nonsterol isoprenoids is attenuated in concomitant with that of cholesterol, which hampers HMGCR protein degradation. Together with enhanced transcription of *HMGCR* as a result of SREBP2 activation, HMGCR protein levels are substantially elevated. This statin-induced compensatory HMGCR increase has been long seen in many species including humans [135–138], which can compromise the efficacy of statins on cholesterol biosynthesis.

Compared with simply occupying the enzymatic active site, elimination of the entire HMGCR protein seems to be a more effective way to block cholesterol biosynthesis. Several small molecule compounds have been developed to induce HMGCR degradation. These include the cholesterol analog with the steroid ring attached by 4,4-dimethyl groups and 7β-hydroxyl group and the side chain by a 1,6-heptadien-4-hydroxy moiety [139], and two proteolysis targeting chimera technology-based molecules composed of a statin at one end and the ubiquitin ligase-recruiting moiety at the other end [140, 141]. In mice, HMGCR degraders alone or together with statins effectively ameliorate diet-induced hypercholesterolemia [139, 141]. Such “degradation-over-inhibition” idea has attracted considerable industry attention and propelled the development of many cancer-targeting molecules that reach clinical stages [142], highlighting the potential of HMGCR degraders on lowering LDL-C. The liver-expressed ubiquitin-specific peptidase 20 (USP20) was recently found to stabilize HMGCR in response to insulin and glucose signals under feeding conditions [143]. Pharmacological inhibition of USP20 can spatiotemporally decrease the HMGCR protein and is therefore beneficial for LDL-C reduction as well.

### Cholesterol catabolism and intestinal microbiota

Cholesterol is eliminated from the body unmetabolized or after conversion to other molecules such as bile acids and coprostanol (Fig. 2). Cholesterol to be excreted includes that from the diet, the bile, and the plasma via the transintestinal cholesterol efflux pathway. Biotransformation to bile acids accounts for about half of the daily turnover of cholesterol [144], and is estimated to be 200–600 mg/day in humans [145]. The bile acid biosynthetic process initiates in the liver, where cholesterol via two major pathways is converted to two primary bile acids and their glycine or taurine conjugates, which are then transported to the gallbladder and secreted as needed into the small intestine [146]. A small population of metabolites further enter the large intestine and colon and are metabolized by a wide variety of microbiota into different secondary bile acids [147]. Most (>95%) of bile acids are reabsorbed and returned to the liver for processing, constituting a cycle of enterohepatic circulation. The remaining 5% of bile acids, which are equivalent in amounts to its daily biosynthesis, are lost in the feces. Bile acid sequestrants have been long used as an adjunct to statin therapy for LDL-C lowering because they can block the reabsorption of bile acids and thus stimulate generation of bile acids from cholesterol, which leads to reductions in hepatic cholesterol levels and upregulation of hepatic LDLR levels. It was recently shown that bile salt hydrolase-active *Lactobacillus reuteri* NCIMB 30242 and VSL#3 (*Lactobacilli*, *Bifidobacteria*, and *Streptococcus salivarius* subsp. *thermophilus*) by deconjugating bile salts reduce bile acid reabsorption, lessen cholesterol solubilization and absorption, and lower LDL-C levels in subjects with hypercholesterolemia [148].

Conversion of cholesterol to coprostanol, a nonabsorbable neutral sterol predominantly present in the feces, represents another major way for cholesterol catabolism and involves the intestinal microbiota as well. This biotransformation can occur via a direct reduction of the C5–C6 double bond of cholesterol, or in three steps with cholestenone and coprostanone formed as the intermediates. More than 10 active intestinal bacterial strains have been isolated from the gastrointestinal tract and feces of many species including humans [149]. A step further, intestinal sterol metabolism A gene (IsmA)-encoding enzyme, a cholesterol-inducible 3β-hydroxysteroid dehydrogenase, was recently found responsible for the first and last steps of microbial cholesterol-to-coprostanol conversion [150]. The presence of IsmA-encoding bacteria was associated with lower total serum cholesterol in over a thousand human subjects across the world, with the magnitude of decrease even larger than that caused by genetic variations of HMGCR and PCSK9 [150]. Identification of more cholesterol-metabolizing microbes or microbial enzymes is of great translational value. Targeting intestinal microbiota will provide a new avenue to lower LDL-C.

## Conclusion

It has been 35 years since lovastatin was approved as the first statin for clinical use (Fig. 1). From then on, other types of statins were rapidly developed and introduced to the market. Due to their remarkable efficacy on lowering LDL-C levels, statins are the most frequently prescribed lipid-lowering drugs worldwide, making them a legendary blockbuster in the history of medicine. Atorvastatin (Lipitor^®^) has brought for Pfizer over $125 billion of revenue during its patent period and continues to generate roughly $2 billion per year. Despite powerful, statins are not the solution for all—some patients may not tolerate statins well or achieve LDL-C target levels even with maximal doses of statins, which calls for the discovery and development of other LDL-C-lowering therapies.

As discussed in this review, enormous progress has been made in developing new LDL-C-lowering therapies (Fig. 1), which reflects a great medical need for treating ASCVD. These include ezetimibe that targets intestinal cholesterol absorption; evolocumab, alirocumab, and inclisiran that target LDL uptake; mipomersen and lomitapide that target VLDL production; evinacumab, vupanorsen, anacetrapib, and obicetrapib that target lipoprotein metabolism; and bempedoic acid that, like statins, targets cholesterol biosynthesis and LDL uptake. While not all reach the commercialization stage, many, such as ezetimibe, PCSK9 mAbs, and lomitapide, have already produced great profits for pharmaceutical companies and, more importantly, provided potent alternatives for patients who are statin intolerant or require additional LDL-C lowering. The development of LDLR-independent therapies—lomitapide and evinacumab—offers unique solutions for homozygous FH patients.

However, this is definitely not the end. More promising LDL-C-lowering strategies are under way as a result of breakthroughs in basic research and technology. HMGCR degraders have been synthesized to overcome the compensatory increases of HMGCR proteins induced by statins. Linking intestinal microbiota to cholesterol metabolism opens up new ways for prevention, diagnosis, and treatment of hypercholesterolemia. With respect to PCSK9 inhibition, multiple novel strategies, including vaccines, nonantibody-binding proteins, mimetic peptides, and CRISPR/Cas9-mediated gene editing, are currently at different stages of development [151]. Various modalities to inactivate ANGPTL3 have been proposed as well [152]. Other than state-of-the-art approaches, novel candidates involved in LDL-C regulation are emerging from recent studies. For example, plasma prekallikrein is a newly identified hepatokine that functions independently of PCSK9 to induce LDLR degradation, and genetic ablation or antibody neutralization of prekallikrein effectively reduces LDL-C levels as well as prevents atherosclerosis in multiple rodent models [153]. LIMA1 has been shown to regulate NPC1L1-dependent intestinal cholesterol absorption, and its loss-of-function mutations cause low LDL-C levels in Chinese Kazakhs [28]. It is hoped that with all these amazing achievements in science and technology better and more effective drugs for lowering LDL-C will be developed in the near future.

Last but not least, although LDL-C is a primary driver for ASCVD and lowering LDL-C is the first priority for preventing and treating ASCVD [4], elevated levels of lipoprotein(a) and TRLs are risk factors as well. Some of the aforementioned LDL-C-lowering therapies, e.g. PCSK9 inhibitors, lomitapide, mipomersen, and CETP inhibitor, can reduce lipoprotein(a) levels [154]. ANGPTL3 inactivation is also the therapy for lowering TRLs [155]. However, for those ineffective on lipoprotein(a) or TRL levels, such as statins and ezetimibe, additional strategies targeting lipoprotein(a) (e.g. pelacarsen and olpasiran) or TRLs (e.g. pemafibrate and volanesorsen) could be taken into account in reducing the risk of ASCVD.
